# Supplementary material for: Liraglutide Reduces Liver Steatosis and Improves Metabolic Indices in Obese Patients Without Diabetes: A 3-Month Prospective Study
Source: Int J Mol Sci. 2025 Jun 19;26(12):5883. doi: 10.3390/ijms26125883 (PMC12192618; doi:10.3390/ijms26125883)
Supplement: Supplementary file 1 [file ijms-26-05883-s001.zip › ijms-3703693-supplementary.pdf]

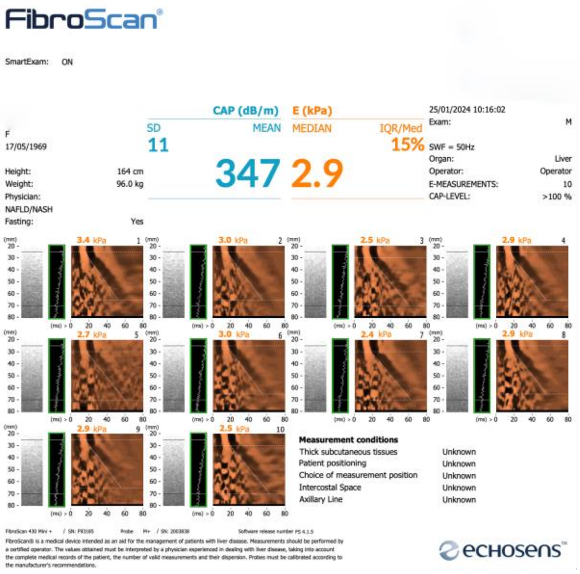

(**a**)

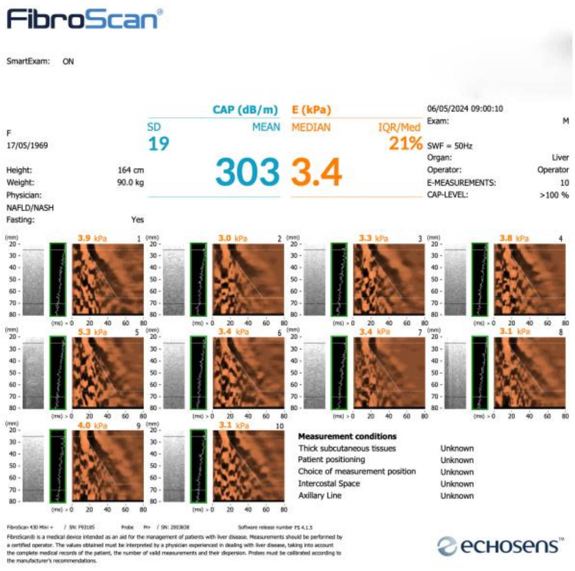

(**b**)

Figure S1. Printouts of the FibroScan examination representative results. (**a**) Printout of the FibroScan examination result before the liraglutide treatment (**b**) Printout of the FibroScan examination result after the liraglutide treatment.
